# Supplementary material for: The quest for identifiability in human functional connectomes
Source: Sci Rep. 2018 May 29;8:8254. doi: 10.1038/s41598-018-25089-1 (PMC5973945; doi:10.1038/s41598-018-25089-1)
Supplement: Supplementary file 1 — Supplementary Information [file 41598_2018_25089_MOESM1_ESM.pdf]

The quest for identifiability in human functional connectomes

Enrico Amico[1,2] and Joaquín Goñi [1,2,3,*]
[1] School of Industrial Engineering, Purdue University, West-Lafayette, IN, USA
[2] Purdue Institute for Integrative Neuroscience, Purdue University, West-Lafayette, IN, USA
[3] Weldon School of Biomedical Engineering, Purdue University, West-Lafayette, IN, USA

Supplementary Information

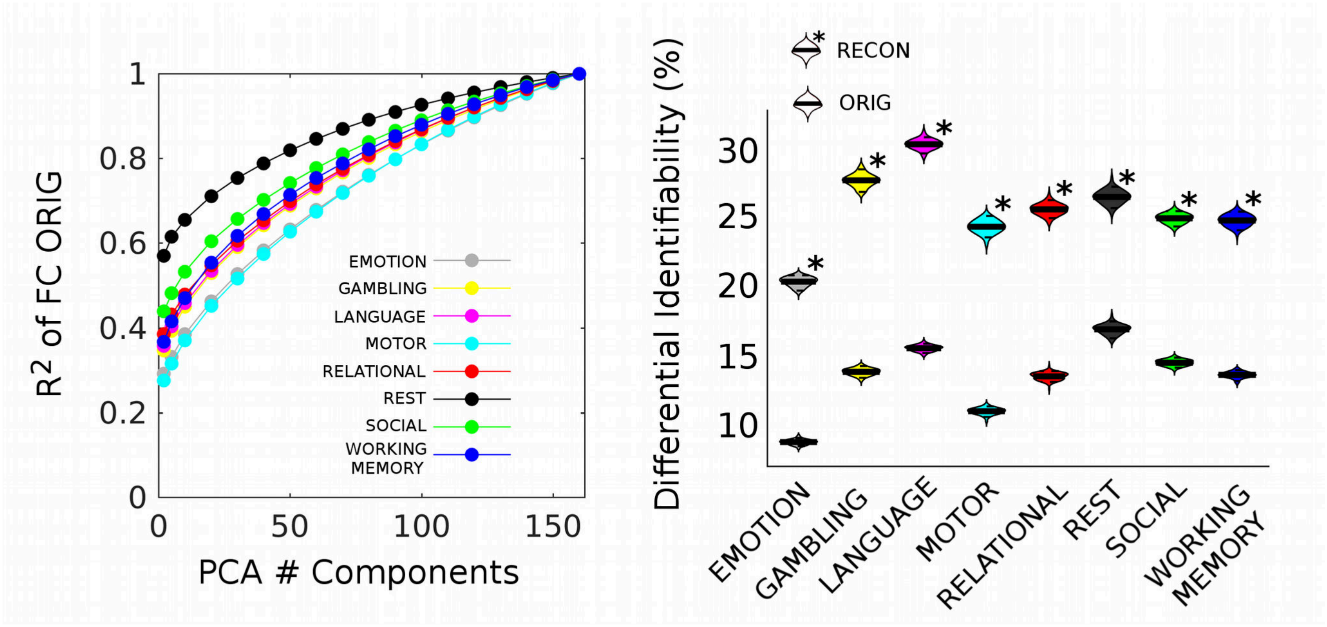

Figure S1. Explained variance and differential identifiability (I_{diff}) across sessions. Left: the variance explained (R-square) of the original data from the PCA reconstruction, for different number of PCA components employed. Each session is plotted with a different color. Right: violin plots show the distribution of the FC individual identifiability (see Methods) across subjects, for each fMRI session (each one has a distinct color), before and after PCA reconstruction. The solid black lines of the violins depict the mean value of the distribution. The asterisk indicates the individual identifiability distributions after reconstruction. Note how the PCA reconstruction always improves the individual identifiability.

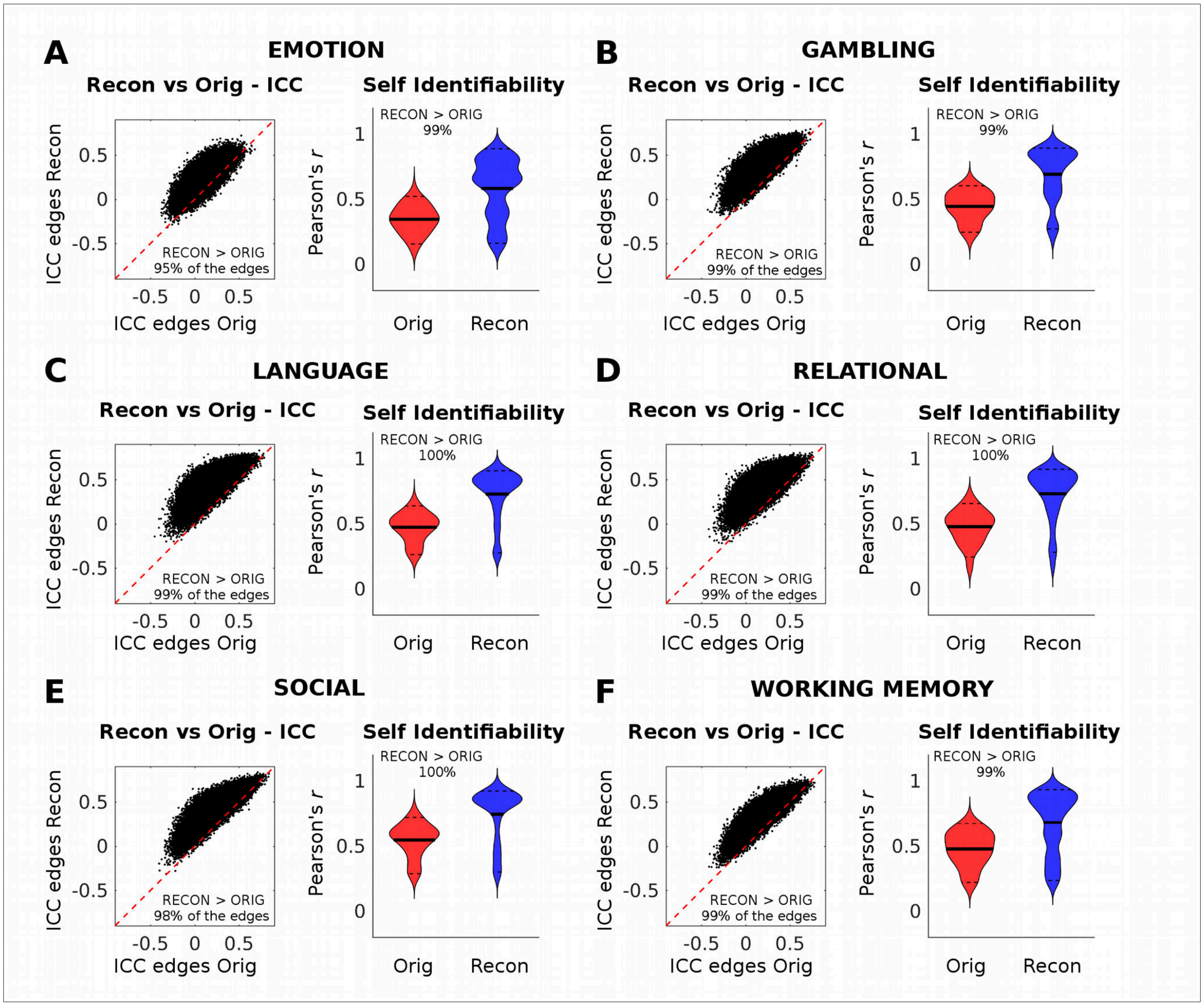

Figure S2. Summary of results on ICC and self-identifiability (I_{self}) for the six fMRI tasks not shown in the main text. Left: the scatter plot edge by edge of the reconstructed ICC values (y axis) vs original ICC values(x axis). The inset reports the percentage of edges where ICC increased after reconstruction (top of the red dashed line) from those that did not (low of the red line). Right: violin plot of the “self identifiability” (i.e., the main diagonal of the identifiability matrix, see Methods) distribution across the 80 subjects, for original (ORIG, red) and reconstructed (RECON, blue). The solid black lines depict the mean value of the distribution; the dashed black lines the 5 and 95 percentiles. The inset specifies the percentage of subjects whose identifiability has improved after PCA reconstruction.

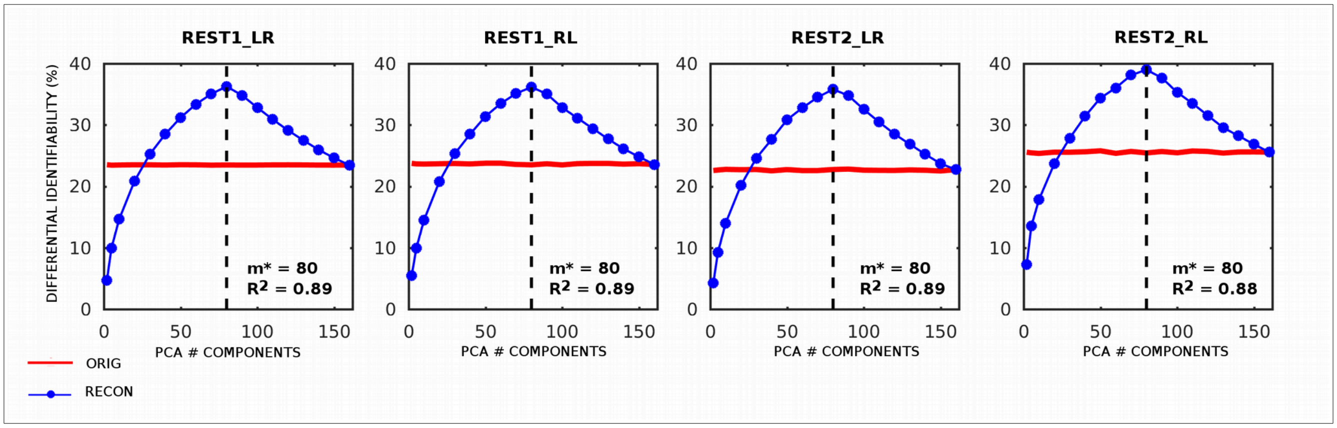

Figure S3. Percent difference of the differential identifiability (I_{diff}) as a function of the number of PCA components used for reconstruction in split resting-state sessions. Plots show, for each split resting state sessions (test = first 600 fMRI frames, retest = second 600 fMRI frames, see Methods for details), I_{diff} as a function of the number of PCA components used for reconstruction (evaluated at 2, 5, and 10 to 160 components in steps of 10). Red line denotes I_{diff} for the original FCs, whereas blue line with circles denotes the identifiability for reconstructed FCs based on the different number of components sampled. For each subplot, the optimal number of components that maximizes differential identifiability (m*) and the corresponding explained variance (R2) are shown. To test the stability of the method, I_{diff} was evaluated over 100 different runs. At each run, 80 subjects were randomly sampled from the HCP resting-state data pool of 100 unrelated subjects, 4 sessions (REST1_LR, REST1_RL, REST2_LR and REST2_RL) for a total of 160 FCs at every run. The standard deviation of I_{diff} (not shown in the plots) across runs was always lower then 0.9 %, for all the sessions considered, for both original and reconstructed data.

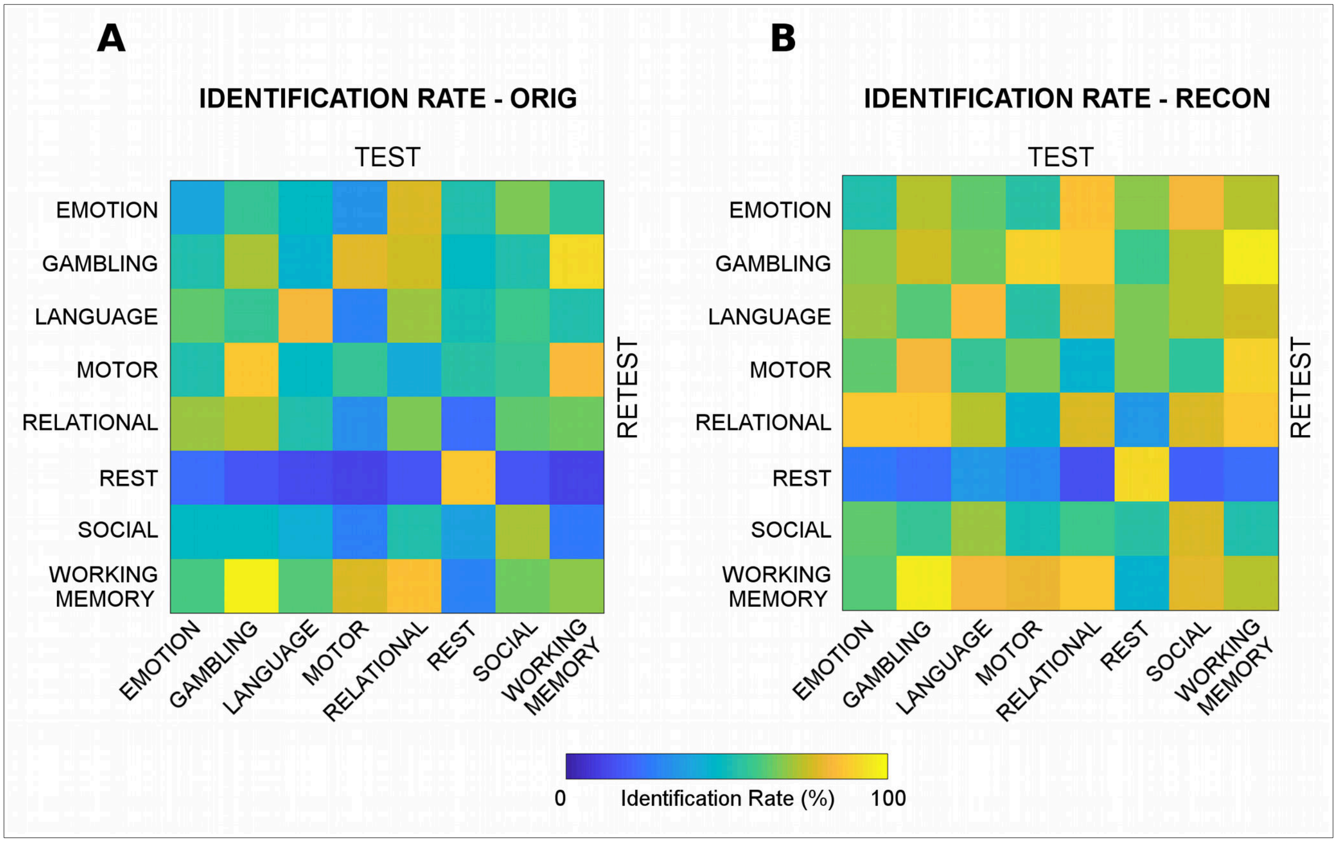

Figure S4: Identifiability rates for all possible combinations of test-retest (within the same sequence and between different sequences), before and after PCA reconstruction. Note that identifiability rates in the main diagonal (i.e. test-retest from the same sequence) were the average of test-retest and retest-test rates. Also, for main diagonal values, the optimal number of PCA components m* was based on the findings of Fig. 2. For off diagonal values, where FCs come from different tasks (T_i,T_j), optimal reconstruction on the mixed data matrix was defined as $m^*_{mixed} = max(m^*_{Ti}, m^*_{Tj})$.

	EMOTION	GAMBLING	LANGUAGE	MOTOR	RELATIONAL	REST	SOCIAL	WORKING MEMORY
**Abs_RMS - μ ± σ**	0.36 ± 0.39	0.39 ± 0.44	0.44 ± 0.45	0.49 ± 0.33	0.43 ± 0.46	0.82 ± 0.44	0.37 ± 0.32	0.56 ± 0.52
**RT (ms) - μ ± σ**	798 ± 143	418 ± 116	359 ± 350	n.a.	1762 ± 327	n.a	1103 ± 358	884 ± 149
**ACC (%) μ ± σ**	98 ± 4	n.a.	88 ± 9	n.a.	76 ± 14	n.a.	n.a	86 ± 10

Table S1: The summary statistic (mean and standard deviation across the 100 unrelated subjects) for the motion and behavioral variables employed in Fig.S5 and Table1, respectively, for each of the fMRI task and resting-state. In order from top to bottom row: absolute frame displacement (Abs_RMS, unitless); task response time (RT, milliseconds); task accuracy (ACC, percentage).

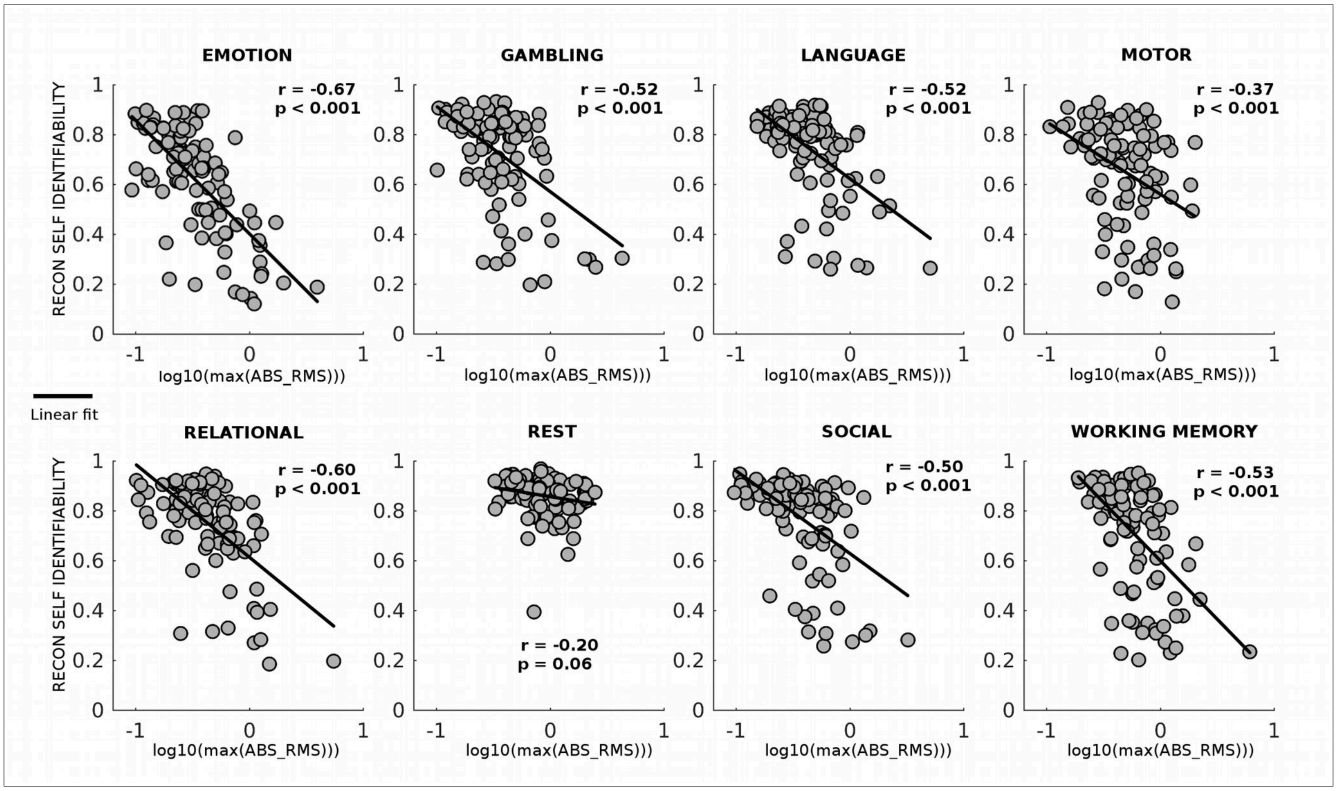

Figure S5. Log-linear trend evaluation between self identifiability (I_{self}) and mean absolute frame displacement. Plot shows, for each resting-state and task session, the scatter plot between individual self identifiability (see Methods for details) values after reconstruction (y-axis) and the log_{10} of the maximum value (across the two sessions) of the average absolute frame displacement (ABS_RMS, x-axis). Solid lines show the linear fit of the scatter plots, and the insets report Pearson's correlation coefficient (r) between these two variables, with the associated significance (p-value). Note how there is a significant negative correlation (p<0.001) between increases in self identifiability and ABS_RMS across all tasks. No significant linear trend is present for the REST acquisition.
